# Cat Ownership Perception and Caretaking Explored in an Internet Survey of People Associated with Cats

**DOI:** 10.1371/journal.pone.0133293

**Published:** 2015-07-28

**Authors:** Sarah Zito, Dianne Vankan, Pauleen Bennett, Mandy Paterson, Clive J. C. Phillips

**Affiliations:** 1 Centre for Animal Welfare and Ethics, School of Veterinary Science, University of Queensland, Gatton Campus, Gatton, QLD, Australia; 2 School of Veterinary Science, University of Queensland, Gatton Campus, Gatton, QLD, Australia; 3 School of Psychology and Public Health, La Trobe University, Bendigo, Victoria, Australia; 4 RSPCA Queensland, Wacol, QLD, Australia; ETH Zurich, SWITZERLAND

## Abstract

People who feed cats that they do not perceive they own (sometimes called semi-owners) are thought to make a considerable contribution to unwanted cat numbers because the cats they support are generally not sterilized. Understanding people’s perception of cat ownership and the psychology underlying cat semi-ownership could inform approaches to mitigate the negative effects of cat semi-ownership. The primary aims of this study were to investigate cat ownership perception and to examine its association with human-cat interactions and caretaking behaviours. A secondary aim was to evaluate a definition of cat semi-ownership (including an association time of ≥1 month and frequent feeding), revised from a previous definition proposed in the literature to distinguish cat semi-ownership from casual interactions with unowned cats. Cat owners and semi-owners displayed similar types of interactions and caretaking behaviours. Nevertheless, caretaking behaviours were more commonly displayed towards owned cats than semi-owned cats, and semi-owned cats were more likely to have produced kittens (p<0.01). All interactions and caretaking behaviours were more likely to be displayed towards cats in semi-ownership relationships compared to casual interaction relationships. Determinants of cat ownership perception were identified (p<0.05) and included association time, attachment, perceived cat friendliness and health, and feelings about unowned cats, including the acceptability of feeding unowned cats. Encouraging semi-owners to have the cats they care for sterilized may assist in reducing the number of unwanted kittens and could be a valuable alternative to trying to prevent semi-ownership entirely. Highly accessible semi-owner “gatekeepers” could help to deliver education messages and facilitate the provision of cat sterilization services to semi-owners. This research enabled semi-ownership to be distinguished from casual interaction relationships and can assist welfare and government agencies to identify cat semi-owners in order to develop strategies to address this source of unwanted cats.

## Introduction

There are large numbers of unwanted cats in many communities. This is a serious problem as many thousands of unwanted cats are euthanized every year [1], unwanted cats often experience poor welfare [2], and management of unwanted cats results in considerable costs to the community [1,3]. Many researchers have identified that humans providing resources, particularly food, to unsterilized and unconfined cats they do not own, promotes excess breeding and is likely to contribute substantially to the unwanted cat problem [2,4,5,6]. People who engage in this activity with specific cats are referred to by some authors as “cat semi-owners” [2,7]. Toukhsati et al. [2] define semi-owners as people who feed or provide care to a cat for which they do not perceive ownership. Cat semi-ownership in the community is reported to be relatively common (10–22%) in many countries including Australia [2], Ireland [8], Italy [9], Thailand [5] and the USA [4,10] and is thought to make a considerable contribution to the creation and maintenance of unwanted cat populations although direct evidence of this has not been reported [2,5,11]. A number of authors have also reported on the contribution of cat semi-ownership to cats entering shelters [1,12,13]. A recent Australian study identified that a third of cats surrendered to the participating animal shelters as “stray” were, in fact, semi-owned [14]. In another Australian study it was suggested that a large proportion of “stray” cats surrendered to the study shelter by the general public were semi-owned based on their weight and sociability (approximately 82% of “stray” cats were of optimal weight or overweight and approximately a quarter of “stray” cats scored 4 or 5 on the Monash Feline Sociability Rating indicating moderate to high sociability) [12].

Semi-owners reportedly display a range of interaction and caretaking behaviours towards their semi-owned cat(s), varying from irregular feeding and few interactions to regular feeding and many interactions [2]. In some instances, interaction and caretaking behaviours of semi-owners may be indistinguishable from behaviours exhibited by people who do perceive themselves as owners [2]. The current definition of semi-ownership does not attempt to distinguish between people who provide short-term or limited support for unowned cats and those who provide regular and ongoing support. This may make identification of semi-owners difficult.

Although people’s perception of ownership and caretaking behaviours are central to understanding cat semi-ownership and its impact on the unwanted cat problem, these have not been investigated in depth. An improved understanding of determinants of cat ownership perception and how cat ownership perception is associated with human-cat interactions and caretaking behaviours may inform the design of policies targeting semi-owners in an effort to minimise the negative effects of this practice.

The Theory of Planned Behaviour [15,16] suggests that a person’s underlying psychosocial characteristics influence their behaviour. This theory has been used to identify psychosocial factors that predict behaviours relating to cat and dog caretaking [7,17,18]. For example, a recent study in Thailand used the Theory of Planned Behaviour to identify religious beliefs, attitudes, social norms and perceived behavioural control factors that predicted the intention to sterilise semi-owned cats and dogs [5]. This study found a negative impact on the intentions to sterilise if sterilisation was perceived to be inconsistent with religious beliefs. The results were then used to recommend strategies to encourage sterilisation of semi-owned cats and dogs, such as education programs emphasising the alignment of responsible ownership practices with cultural and religious beliefs and values [5]. The Theory of Planned Behaviour has also been used to identify beliefs associated with improved owner compliance with responsible dog management practices, which led to the recommendation that appropriate role models be used in advertising campaigns [17]. It was postulated that the Theory of Planned Behaviour could be used to identify factors associated with cat ownership perception.

Online surveys that employ a “virtual snowballing” sampling technique, whereby a recruitment email is sent to an initial group of people who are asked to forward information about the survey to their social networks, can allow inexpensive and rapid collection of data from a large number of people. The anonymity of this sampling technique may also decrease the likelihood of false reporting [19] and provide access to people, such as cat semi-owners, who are generally difficult to access. For this reason, it has been used by others in similar circumstances [19,20,21,22,23,24,25,26,27]. Although the data obtained through internet surveys may be prone to selection bias and it is not possible to estimate response rates, internet surveys can result in findings consistent with traditional sampling methods and make valuable contributions to research in fields such as psychology [26]. This approach may also be valuable for enhancing our understanding of cat ownership perception.

Previously aspects of cat ownership perception in people surrendering cats to Australian RSPCA shelters have been investigated [14]. In this previous study, interactions with surrendered cats were described and association time and acquisition mode were identified as key determinants of cat ownership perception in cat surrenderers. Strong associations were found between perception of cat ownership, human-cat interactions and caretaking, and some evidence of associations between attitudes and cat ownership perception was also found. While this previous study involved only people who surrendered cats, it was postulated that these results may also be relevant in a larger sample of the general population and that the Theory of Planned Behaviour could be used to identify further determinants of cat ownership perception in the current study.

It was hypothesised that cat owners, semi-owners and people who had only casual interactions with cats would have differing human-cat relationships, which would result in differing types and frequencies of cat caretaking behaviours and interactions. Specifically, cat owners were expected to show more types and frequencies of responsible behaviours (such as sterilisation, microchipping, confinement, identification) towards their cats compared to cat semi-owners. The primary objective of the study was to identify key determinants of cat ownership perception and to evaluate how identified determinants are associated with interactions and caretaking behaviours displayed towards the cat. A secondary objective was to validate a more specific definition of cat semi-ownership so that the semi-owners can be distinguished from people who have casual interactions with unowned cats.

## Methods

### Study overview

Data were collected from a convenience sample of self-selected participants who volunteered to complete an online survey between 16^th^ December 2013 and 23^rd^ April 2014. The study, including the consent procedure, was approved by the University of Queensland Ethics Committee (project number 2011001160).

### Participants and recruitment

All people over 18 years of age were eligible to complete the survey which was presented in the English language. Participants were recruited using a “virtual snowballing” technique which involved requesting personal and professional contacts of the research team (by email or through Facebook.com) to complete the survey and to forward this request to their personal and professional contacts. Those who volunteered to participate clicked on a link in the message which linked directly to the survey site. On the first page of the survey participants were shown information about the questionnaire including the following: that participation was voluntary, participants needed to be 18 years of age or older, the study had been cleared in accordance with the ethical review guidelines and processes of The University of Queensland, participants were able to discuss their participation with project staff or the School Ethics officer (contact details were given) and how the information gathered would be used. Respondents were then asked to confirm that they were over 18 years of age, that they understood that their participation in the study was voluntary, consented to participate and to their responses being used as outlined, by clicking on the link to continue to the survey. Only participants who gave consent answered questions from the survey.

Respondents who did not answer questions about a cat or who resided outside of Australia were not enrolled in the study. Respondents with incomplete questionnaires were excluded, as were respondents with questionnaire completion times of <2.5 minutes (the minimum time calculated by the research team to genuinely complete the questionnaire). During the online survey each respondent was able to answer questions for one owned cat, one “stray” (i.e. unowned) cat, or one of each. The term “stray” cat was used throughout the questionnaire to refer to unowned cats as this colloquial term was thought likely to be familiar to most respondents; it was assumed that respondents considered that the term “stray” cat meant unowned and cats designated as “stray” in the questionnaire are referred to as unowned in this paper.

### Sample size calculations

*A priori* statistical power calculations were performed based on numbers of cats required to detect associations between binary demographics, attitude measures, caretaking behaviour measures, and the respondent’s perception of ownership of the cat with which they interacted (owned or unowned) using the Compare 2 module (version 2.69) of WinPepi (version 11.39;[28]). Statistical power was calculated for various total sample sizes, ratios of owned to unowned cats, and assumed proportions exposed (rather than not exposed) for a binary measure for each of owned and unowned cats. Statistical power was calculated for two-sided exact mid p-values; alpha was set at 0.05. These calculations showed that statistical power would be high (above 95%) for detecting absolute differences in proportions for binary outcome measures of 0.1 or more between owned and unowned cats (2/3 owned, 1/3 unowned) if 912 cats were enrolled. Therefore, we aimed to enrol respondents for 1000 cats.

### Questionnaire design and data collection

The questionnaire was developed through an iterative process of literature review, consultations with academic and industry experts, testing for reliability and validity with test subjects (not enrolled participants), and revision. The questionnaire contained both forced choice and open ended questions that interrogated respondent demographics, cat ownership and interaction history, beliefs, attitudes, social norms and perceived behavioural control relating to cats. The latter were developed based on the Theory of Planned Behaviour [15,16] and a previous study that investigated attitudes and behaviours towards cats in the community [2]. Questionnaire details are provided in S1 Table.

Survey respondents were given a brief explanation of the study and instructions on how to begin. Respondents entered answers directly into a digitized questionnaire [29]. Those identifying themselves as cat owners were directed to answer a set of questions for one of their owned cats (the one whose name began with the letter closest to the beginning of the alphabet). Those indicating that they had interacted with one or more unowned cats were asked to answer questions about the unowned cat they had interacted with the most in the past five years.

To explore the relationship between socioeconomic status and perception of cat ownership, the index of relative socioeconomic advantage and disadvantage calculated from 2011 census data was used [30]. This index described the socioeconomic status of the respondent's home area based on postcode; respondents were classified based on the national decile of this index for their home postcode.

### Data analyses

#### Descriptive statistics

All statistical analyses were performed with Stata12 (version 12.1 StataCorp, 4905 Lakeway Drive, College Station, Texas 77845, USA) using the individual cat as the unit of analysis and models that accounted for clustering of cat within respondent. Not all respondents answered every question; proportions are reported as percentages of those respondents who answered each question.

Each cat was categorised into one of four groups based on its relationship with the respondent; these were defined by the respondent’s perception of their ownership of the cat, their length of association with the cat, their frequency of feeding the cat (for non-owners), and their method of acquiring the cat (for owners). These divisions were informed from the results of previous studies [2,14]. Acquisition method was classified as passive or active, depending on whether cat owners reported that they had planned to acquire the cat or not. The four human-cat relationship categories were defined as follows:

1. Casual interaction – a human-cat relationship in which the respondent did not perceive themselves as the owner of the cat, and had interacted with the cat for less than one month and/or had fed the cat only occasionally or not at all
2. Semi-ownership – a human-cat relationship in which the respondent did not perceive themselves as the owner of the cat, but had interacted with the cat for at least a month and had fed the cat frequently or always
3. Ownership of a passively-acquired cat—a human-cat relationship of any duration in which the respondent perceived themselves as the owner of the cat and had acquired the cat passively
4. Ownership of an actively acquired cat—a human-cat relationship of any duration in which the respondent perceived themselves as the owner of the cat and had acquired the cat actively

This definition of semi-ownership is based on previous definitions in the literature [2,5], with the addition of a minimum association time based on previous work showing a strong association between association time and human-cat relationship type [14] and a feeding frequency. One month was chosen for the association time because this association time is arbitrarily used by some shelters to classify an incoming “stray” cat as “owned” (RSPCA staff, personal communication November 2013).

The interactions and caretaking behaviours later compared statistically between the four human-cat relationship categories were purposely not used in the categorization of the cats into the four different relationships.

#### Identification of determinants of ownership perception for semi-owned cats and owned passively acquired cats

Cat ownership perception was only expected to vary within human-cat relationships where the cat was not actively acquired. Since acquisition method is a strong predictor of cat ownership perception, potential determinants of cat ownership perception were only assessed using respondents with semi-owned cats and those with passively-acquired owned cats. The time period for which the respondent had an association with the surrendered cat is also a strong predictor of cat ownership perception [14] and, consequently, could be a major confounder of the results for other potential determinants. Therefore, the analysis of determinants of cat ownership perception was also restricted to only those human-cat relationships where the respondent had been associated with the cat for at least one month and, in addition, analyses of the relationship between independent variables and the respondent’s perception of cat ownership (the dependent variable) were adjusted for association time, fitted as a categorical variable (the categories were 1 month to <6 months, ≥6 months <12 months, ≥1 year < 3 years, and ≥3 years).

All variables measuring respondent demographics, beliefs, attitudes, social norms and perceived behavioural control factors, cat factors, attachment and association time were treated as independent variables and were subjected to initial analyses (adjusted only for association time) to screen for associations with perception of cat ownership (the dependent variable) (Table 1 and S2 Table). Random effects logistic regression was used, with a random effect of respondent fitted to account for clustering of cat within respondent. The-xtlogit- function in Stata was used for this purpose. Likert scale responses (initially quantified using a 5-point Likert scale, which measured agreement with each statement, from “strongly disagree” through “neither agree nor disagree” to “strongly agree”) were collapsed, where necessary, into three or four categories for analysis to avoid sparse or zero category combinations (see Table 1 and S2 Table for details of which independent variables were collapsed). All p-values from the initial analysis were adjusted for multiple tests of significance using the Benjamini-Hochberg Step-up False Discovery Rate method, with the Etcetera module in WinPepi (version 11.11; [28]).

**Table 1:** Distributions of study cats in semi-ownership and ownership (of passively acquired cats) human-cat relationships, potential determinants of ownership perception and associations between these determinants and the perception of ownership of the study cat^1^.

Independent variable and categories	Semi-owned cats	Owned passively-acquired cats	Adjusted odds ratio^2^	95% Confidence interval		P value^3^
	n (% of cats)	n (% of cats)				
**Multivariable model group 1: Beliefs about cats, cat ownership and “stray” cats**						
**Agreement with the statements:**						
**“Cats kill wildlife” (n = 341)** ^**4**^						**0.07**
Strongly disagree	1 (1)	0	Reference category			
Somewhat disagree	0	5 (2)	0.8	0.1 to 10.9		0.88
Neither agree nor disagree	9 (9)	53 (22)	0.4	0.0 to 4.5		0.47
Somewhat agree	63 (64)	148 (61)	0.3	0.0 to 3.3		0.33
Strongly agree	25 (26)	38 (16)	1.0	0.1 to 12.3		1.00
**“Cats are expensive pets” (n = 342)** ^**4**^						**0.04**
Strongly disagree	18 (18)	22 (9)	Reference category			
Somewhat disagree	44 (45)	104 (43)	1.3	0.6 to 3.1		0.55
Neither agree nor disagree	27 (28)	81 (33)	2.4	1.0 to 6.1		0.06
Somewhat or strongly agree	9 (9)	37 (16)	3.7	1.1 to 12.0		0.03
**“Stray cats are a nuisance” (n = 342)** ^**4**^						**0.04**
Strongly disagree	13 (13)	14 (6)	Reference category			
Somewhat disagree	26 (27)	47 (23)	1.7	0.6 to 5.0		0.34
Neither agree nor disagree	32 (33)	57 (23)	1.5	0.5 to 4.5		0.44
Somewhat agree	24 (25)	101 (41)	3.9	1.3 to 11.4		0.01
Strongly agree	3 (3)	25 (10)	2.9	0.5 to 15.2		0.22
**Multivariable model group 2: Attitudes towards cats, cat ownership and “stray” cats**						
**Agreement with the statements:**						
**“Feeding a stray cat is the right thing to do” (n = 342)** ^**4**^						**0.26**
Strongly disagree	5 (5)	35 (14)	Reference category			
Somewhat disagree	7 (7)	49 (20)	3.3	0.7 to 16.4		0.15
Neither agree nor disagree	29 (30)	84 (34)	2.1	0.5 to 9.4		0.31
Somewhat agree	41 (42)	58 (24)	1.2	0.3 to 5.5		0.81
Strongly agree	16 (16)	18 (7)	1.8	0.3 to 9.7		0.52
**“Feeding stray cats stops them from killing wildlife” (n = 342)** ^**4**^						**0.18**
Strongly disagree	7 (1)	52 (21)	Reference category			
Somewhat disagree	33 (34)	86 (35)	0.3	0.1 to 1.0		0.05
Neither agree nor disagree	28 (29)	58 (24)	0.2	0.1 to 0.8		0.02
Somewhat agree	25 (26)	42 (17)	0.3	0.1 to 1.3		0.10
Strongly agree	5 (5)	6 (3)	0.3	0.0 to 2.0		0.20
**“Feeding a stray cat makes me feel good” (n = 341)** ^**4**^						**0.000**
Strongly or somewhat disagree	1 (1)	42 (18)	Reference category			
Neither agree nor disagree	12 (12)	75 (31)	0.2	0.0 to 1.8		0.15
Somewhat agree	50 (51)	98 (40)	0.1	0.0 to 0.5		0.01
Strongly agree	35 (36)	28 (12)	0.0	0.0 to 0.3		0.003
**Multivariable model group 3: Social norms relating to cat ownership and “stray” cats**						
**Agreement with the statements:**						
**“People who are important to me would approve of me feeding a stray cat” (n = 342)** ^**5**^						**0.00286**
Strongly disagree	1 (1)	12 (5)	Reference category			
Somewhat disagree	4 (4)	34 (14)	0.5	0.0 to 5.5		0.57
Neither agree nor disagree	19 (19)	78 (32)	0.4	0.0 to 3.9		0.45
Somewhat agree	33 (34)	81 (33)	0.2	0.0 to 1.9		0.16
Strongly agree	41 (42)	39 (16)	0.1	0.0 to 1.0		0.05
**Multivariable model group 4: Perceived behavioral control relating to cat ownership and “stray” cats**						
**Agreement with the statements:**						
**“I could not feed a stray cat because of my beliefs” (n = 342)** ^**4**^						**0.000**
Strongly disagree	65 (66)	93 (38)	Reference category			
Somewhat disagree	24 (25)	81 (33)	1.8	0.9 to 3.6		0.11
Did not disagree^6^	9 (9)	70 (29)	5.5	2.3 to 13.3		0.000
**“Financially I could afford to feed a stray cat’” (n = 342)** ^**4**^						**0.23**
Strongly disagree	9 (9)	15 (6)	Reference category			
Somewhat disagree	7 (7)	27 (11)	1.4	0.4 to 5.6	0.63	
Neither agree nor disagree	10 (10)	41 (17)	0.9	0.3 to 3.2	0.86	
Somewhat agree	34 (35)	115 (47)	1.3	0.4 to 3.8	0.64	
Strongly agree	38 (39)	46 (19)	0.6	0.2 to 1.7	0.31	
**Multivariable model group 5: Cat factors**						
**Perceived cat friendliness (n = 342)** ^**4**^						**0.000**
Unfriendly	8 (8)	1 (1)	Reference category			
Neither friendly nor unfriendly	28 (29)	13 (5)	3.6	0.3 to 39.0		0.29
Friendly	62 (63)	230 (94)	19.4	2.0 to 188.7		0.01
**Perceived cat health (n = 342)** ^**4**^						**0.000**
Bad	15 (15)	3 (1)	Reference category			
Neither good nor bad	33 (34)	15 (6)	2.9	0.7 to 13.1		0.16
Good	50 (51)	226 (93)	18.9	4.9 to 73.7		0.000
**Association time with the cat (n = 342)** ^**5**^						**0.000**
1 month to <6 months	32 (33)	14 (6)	Reference category			
≥6 months <12 months	14 (14)	21 (9)	3.4	1.4 to 8.6		0.009
≥1 year < 3 years	29 (30)	55 (23)	4.3	2.0 to 9.4		0.000
≥3 years	23 (24)	154 (63)	15.3	7.1 to 32.9		0.000
**Respondent’s attachment to the cat (n = 341)** ^**5**^						**0.000**
(1) Not at all attached	6 (6)	2 (1)	Reference category			
(2)	13 (13)	4 (2)	0.9	0.1 to 6.8		0.89
(3)	26 (27)	19 (8)	2.1	0.3 to 12.6		0.42
(4)	28 (29)	54 (22)	3.9	0.7 to 22.7		0.13
(5) Very attached	24 (25)	165 (68)	13.6	2.4 to 77.1		0.003

^1^ A multivariable model was analysed for each of the five groups described in methods. Independent variables from each of the five groups that had an overall p-value of ≤0.05 on initial screening were included in these multivariable models; all independent variables fitted in those models are reported in this table

^2^ Odds ratio estimates were adjusted for association time and for all other independent variables reported in this table. Odds ratios refer to the odds of a cat having an ownership human-cat relationship compared to a semi-ownership human-cat relationship.

^3^ Bold values are overall likelihood ratio test p-values for the independent variable; non-bolded values are Wald p-values for the specific category, relative to the reference category.

^4^ Odds ratio, confidence interval and p value derived from the multivariable analysis for the independent variable’s category; 341 cats were included in the multivariable model for the belief and attitude groups, 342 for the perceived behavioural control and cat factors groups; this may be less than the total numbers shown for each independent variable as cats without missing values for any of these independent variables were excluded from the multivariable model.

^5^ Odds ratio, confidence interval and p value derived from the initial screening analysis of that independent variable

^6^ Includes “neither agree nor disagree”, “somewhat agree” and “strongly agree”.

To remove any potential confounding effect of other measured variables, multivariable models were then fitted with the independent variables that were significantly different (i.e. p-value adjusted for multiple tests of significance ≤0.05) between semi-owners and owners in the initial screening analyses. Due to the limits of the sample size, complex multivariable models that included all significant independent variables could not be fitted. Therefore, each independent variable (potential determinant) was allocated into one of five groups; first the independent variables based on the Theory of Planned Behaviour were allocated to four groups based on the Theory’s categories (beliefs, attitudes, social norms and perceived behavioural control), then a fifth group was created to analyse cat factors (perceived cat friendliness and health). A separate multivariable model containing every significant independent variable from each given group, along with association time, was then created for each of the five groups. The results from these multivariable models are reported in Table 1. “Attachment to the cat” and “association time”, were analysed individually because they did not fit into any group and these individual model results are also reported in Table 1. Results for variables that had an overall p-value of >0.05 in the initial screening are reported in S2 Table; the data reported are those from the univariable screening analysis model adjusted for association time.

#### Associations between perception of cat ownership and interactions and caretaking behaviours displayed towards cats

Associations between perception of cat ownership and each interaction and caretaking behaviour displayed toward the study cat were assessed using only semi-owned cats and owned passively-acquired cats that the respondent had been associated with for at least one month. In these comparisons each variable measuring an interaction or caretaking behaviour was treated as a dependent variable and was compared in univariable analyses between human-cat relationships (semi-ownership or ownership of a passively acquired cat), which were treated as the independent variable. Associations between the independent variable (perception of cat ownership) and dependent variables (interactions and caretaking behaviours) were assessed using regression models. Distributions of people’s responses for each binary dependent variable were compared between independent variables using random effects logistic regression in order to account for clustering of responses by respondent, using the-xtlogit- command in Stata. Distributions of dependent variables with more than two outcome possibilities were compared for each independent variable using proportional odds models, with the-ologit- command in Stata. Robust standard errors that accounted for clustering of cat by respondent were used. The exponentiated coefficients from these models estimated the effect of each independent variable on the odds of each dependent variable outcome possibility. These estimates are based on the assumption that the ratio of the odds is the same regardless of which value of the outcome is used as a cutpoint (the proportional odds assumption). For each outcome, this assumption was checked by comparing the log-likelihoods of the proportional odds model and the corresponding multinomial logit model, using the likelihood ratio test without accounting for clustering of cat with respondent. Non-proportional odds were evident for six dependent variables as indicated by a low p-value from likelihood ratio test [≤0.05]), and results from the multinomial logistic model were used for these dependent variables, rather than from the proportional odds model, with robust standard errors that accounted for clustering of cat with respondent.

#### Associations of casual interaction and semi-ownership human-cat relationships with interactions and caretaking behaviours displayed towards study cats

Using the methods described above, the association of the casual interaction and cat semi-ownership human-cat relationship categories with each of the interactions and caretaking behaviours displayed towards the study cat were assessed. In these comparisons each variable measuring an interaction or caretaking behaviour was treated as a dependent variable and was compared in univariable analyses between human-cat relationships (casual interaction or semi-ownership) which were treated as the independent variable.

Associations between the independent variable (casual interaction or cat semi-ownership) and dependent variables (interactions and caretaking behaviours) were assessed using logistic regression. There was no need to account for clustering of cat by respondent because no respondents were included in both casual interaction and cat semi-ownership human-cat relationship categories.

## Results

### Descriptive statistics

A total of 2188 respondents met the predetermined selection criteria; of these, 623 were non-Australian respondents and so were not enrolled. Of the 1565 eligible respondents that began the survey, respondents with incomplete questionnaires (n = 6) and respondents with questionnaire durations of less than 2.5 minutes (n = 63) were excluded, leaving a total of 1496 respondents. Of these, 483 did not answer any cat specific questions (because they did not own a cat or interact with an unowned cat) and 1013 respondents answered questions about at least one specific cat (the “study respondents”). These respondents provided data for 1305 individual cats (“study cats”); 562 respondents provided data for one owned cat, 159 for one unowned cat and 584 for both one owned and one unowned cat. Of these 1305 study cats, for 353 their human-cat relationship were classified as a casual interaction, 98 as semi-ownership, 249 as ownership of a passively-acquired cat and 605 as ownership of an actively-acquired cat. Of the 98 cat semi-owners, 84% (82) were also cat owners, and of the 353 respondents who had a casual interaction with an unowned cat, 59% were also cat owners (210).

Of the 1013 study respondents, most were female (85%; 861/1013), almost one half lived in a suburban location (49%; 498/1013), the median age was 41 years, the median of the respondents’ Index of relative socio-economic advantage disadvantage decile values was 8 (range 1–10 and a higher number indicates relative advantage), and almost one third were educated at university undergraduate degree (30%; 300/1011) or post-graduate degree (31%; 329/1011) level. Approximately half of the respondents were employed full-time (51%; 516/1013), had a combined annual household income before taxes that they rated as being average relative to other households in their country of residence (52%; 519/1004), and most (64%; 622/969) did not follow any religion.

**Fig 1:**
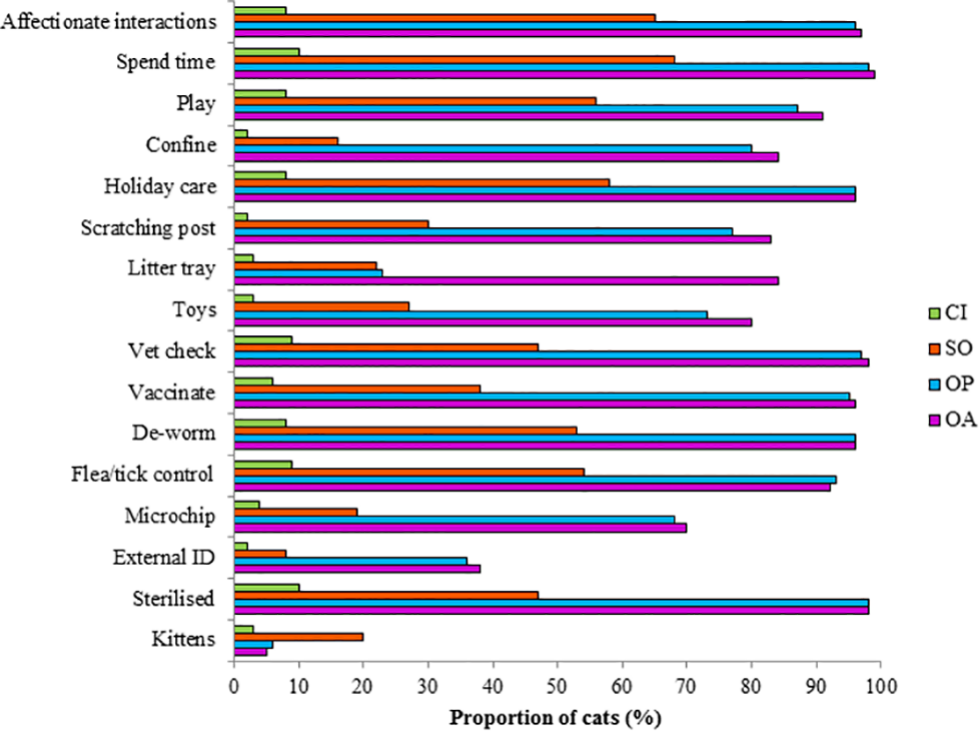
Interactions with and caretaking of study cats in four human-cat relationship categories. Proportions of 1305 study cats in various human-cat relationship categories that were reported to have had kittens, been sterilized, been microchipped, and had received various interactions and caretaking behaviours. CI = casual interaction, SO = semi-ownership, OP = ownership of a passively acquired cat and OA **=** ownership of an actively acquired cat. Flea/tick control, de-worm, vaccinate and vet check proportion includes those cats that received these caretaking behaviours occasionally or regularly. Toys, litter tray and scratching post proportion includes those cats for which these were provided often or always. Holiday care proportion includes those cats for which holiday care was organized sometimes or always. Confine, play, spend time and affectionate interactions (holding, stroking, and cuddling the cat) proportion includes those cats that received these interactions or caretaking behaviours sometimes or daily. ID = identification.

Interactions and caretaking behaviours displayed by respondents from the four human-cat relationship categories are presented in Fig 1. Notably, the majority of owners (of both passively- and actively-acquired cats) displayed most interactions and caretaking behaviours towards their cat, and 98% of owned cats (passively- and actively-acquired owned cats pooled) were sterilized, with owners being responsible for the sterilization in 58% of cases. Many semi-owners also displayed interaction and caretaking behaviours towards their cat and 47% of semi-owned cats were sterilized, with semi-owners being responsible for the sterilization in 58% of cases. By contrast, interactions and caretaking behaviours were rare for casual interaction cats. The majority of owners indicated that they were attached to their cat (94%; 781/837), as did many semi-owners (54%; 52/98), while few people who had a casual interaction with the cat felt attached to the cat (5%; 15/341).

### Identification of determinants of perception of cat ownership for semi-owned cats and owned passively-acquired cats

Results for significant and non-significant independent variables are reported in Table 1 and S2 Table, respectively. The results reported for independent variables that had significant differences between semi-owned cats and owned passively-acquired cats are those from the multivariable models except for “attachment to the cat” and “association time” which were not included in a group multivariable model, so results from initial analysis only are reported for these two variables. The results reported for independent variables that had non-significant differences between semi-owned cats and owned passively-acquired cats are those from the univariable screening models adjusted for association time. Agreement with the statement “people important to me would approve of me feeding a stray cat” was the only significant independent variable in the social norms group and so results from initial analysis only are reported for this independent variable.

Within semi-owned and passively-acquired owned cats, perception of cat ownership was more likely when the respondent was more attached to the cat, when the respondent had been associated with the cat for a longer time, when the respondent felt that they could not feed a stray cat because of their beliefs and thought that stray cats were a nuisance and that cats were expensive pets. Perception of cat ownership was more likely for cats that had a friendly disposition and were healthy. Perception of cat ownership was less likely for respondents for whom feeding a stray cat made them feel good, and respondents who anticipated approval from other important people in their lives for feeding a stray cat (Table 1).

### Associations between perception of cat ownership and interactions and caretaking behaviours displayed towards semi-owned cats and owned passively-acquired cats

All interactions and caretaking behaviours were significantly more likely to be displayed towards cats perceived as owned compared to cats in semi-ownership relationships (p<0.01) (Table 2). Semi-owned cats were more likely than passively-acquired owned cats to have had kittens (p<0.01).

**Table 2:** Distributions of study cats in semi-ownership and ownership (of passively-acquired cats) human-cat relationships that had received various interactions and caretaking behaviours and associations between these and perception of ownership of the study cat.

Dependent variable and categories	Semi-owned cats	Owned passively-acquired cats	Odds ratio/relative risk ratio^1^	95% Confidence interval	P value^2^
	n (% of cats)	n (% of cats)			
**Held/stroked/cuddled cat (n = 342)** ^**3**^			15.0	8.5 to 26.4	**0.000**
Never or occasionally	35 (36)^4^	9 (4) ^4^			
Sometimes	27 (28)	16 (7)			
Daily	36 (37)	219 (90)			
**Spent time with cat (n = 342)** ^**3**^			14.0	7.7 to 25.2	**0.000**
Never	32 (33)	7 (3)			
Sometimes	23 (24)	14 (6)			
Daily	43 (44)	223 (91)			
**Played with cat (n = 342)** ^**5**^					**0.000**
Never	30 (31)	2 (1)	Base outcome		
Occasionally	14 (14)	32 (13)	34.3	7.2 to 162.3	0.000
Sometimes	26 (27)	52 (21)	30.0	6.6 to 135.6	0.000
Daily	28 (29)	158 (65)	84.6	19.1 to 375.3	0.000
**Cat was confined to the respondent’s property (n = 342)** ^**3**^			21.4	12.2 to 37.4	**0.000**
Never	76 (78)	33 (14)			
Occasionally	6 (6)	16 (7)			
Sometimes	10 (10)	25 (10)			
Daily	10 (10)	170 (70)			
**Organized holiday care for cat (n = 342)** ^**5**^					**0.000**
Never	41 (42)	10 (4)	Base outcome		
Sometimes	14 (14)	21 (9)	6.2	2.4 to 15.7	0.000
Always	43 (44)	213 (87)	20.3	9.7 to 42.4	0.000
**Provided a scratching post for cat (n = 342)** ^**3**^			8.5	5.2 to 14.0	**0.000**
Never	65 (66)	45 (18)			
Occasionally	4 (4)	10 (4)			
Sometimes	4 (4)	8 (3)			
Always	25 (26)	181 (75)			
**Provided a litter tray for cat (n = 342)** ^**3**^			16.8	9.8 to 29.0	**0.000**
Never	69 (70)	29 (12)			
Occasionally	7 (7)	12 (5)			
Sometimes	1 (1)	5 (2)			
Always	21 (21)	198 (81)			
**Provided toys for cat (n = 342)** ^**5**^					**0.000**
Never	55 (56)	21 (9)	Base outcome		
Occasionally	16 (16)	47 (19)	7.7	3.6 to 16.3	0.000
Sometimes	8 (8)	34 (14)	11.1	4.5 to 27.6	0.000
Always	19 (19)	142 (58)	19.6	9.8 to 39.1	0.000
**Had cat checked by a veterinarian (n = 342)** ^**5**^					**0.000**
Never	52 (53)	7 (3)	Base outcome		
Occasionally	28 (29)	76 (31)	20.2	8.2 to 49.6	0.000
Regularly	18 (18)	161 (66)	66.4	26.4 to 167.2	0.000
**Had cat vaccinated (n = 342)** ^**5**^					**0.000**
Never	61 (62)	14 (6)	Base outcome		
Occasionally	13 (13)	56 (23)	18.8	8.2 to 43.1	0.000
Regularly	24 (25)	174 (71)	31.6	15.4 to 64.9	0.000
**Gave cat deworming medication (n = 342)** ^**5**^					**0.000**
Never	47 (48)	10 (4)	Base outcome		
Occasionally	23 (24)	57 (23)	11.7	5.1 to 26.6	0.000
Regularly	28 (29)	177 (73)	29.7	13.6 to 64.7	0.000
**Applied flea/tick control to cat (n = 342)** ^**5**^					**0.000**
Never	46 (47)	20 (8)	Base outcome		
Occasionally	25 (26)	66 (27)	6.1	3.1 to 12.1	0.000
Regularly	27 (28)	158 (65)	13.5	7.0 to 26.1	0.000
**Cat had been microchipped (n = 336)** ^**6**^					**0.000**
No	75 (81)	40 (20)	Reference category		
Yes	18 (19)	166 (81)	6.30e+13	1.53e+13 to 2.59e+14	0.000
Don’t know^7^	4	33			
**A tag had been put on cat with the respondent’s contact details (external identification) (n = 340)** ^**6**^					**0.0044**
No	89 (92)	156 (64)	Base outcome		
Yes	8 (8)	87 (36)	10.3	2.1 to 51.8	0.004
**Cat had been sterilized (n = 342)** ^**6**^					**0.000**
No	19 (29)	2 (1)	Reference category		
Yes	46 (71)	240 (99)	49.6	11.2 to 219.9	0.000
Don’t know^7^	33	2			
**Cat had had kittens (n = 342)** ^**6**^					**0.000**
No	38 (70)	210 (93)	Reference category		
Yes	16 (30)	15 (7)	0.2	0.1 to 0.4	0.000
Don’t know^7^	44	19			

^1^ Odds ratio estimates are reported for ordered logistic regression and random-effects logistic regression; these estimate the odds of any particular interaction or caretaking behaviour outcome category for a cat with an ownership human-cat relationship compared to those cats with a semi-ownership human-cat relationship. Relative risk ratio (RRR) estimates are reported for multinomial logistic regression analyses; these estimate the probability of the specified interaction or caretaking behaviour outcome category rather than the base outcome for cats with an ownership human-cat relationship compared to those cats with a semi-ownership human-cat relationship

^2^ Bold values are overall likelihood ratio test p-values; non-bolded values are Wald p-values for the specific level, relative to the reference category

^3^ Results from ordered logistic regression as >2 categories for outcome (adjusted for clustering by respondent) and there was no evidence that odds were not proportional.

^4^ Total numbers of respondents differ between variables as not all respondents answered each question and, within variables, percentages do not always sum to 100% due to rounding

^5^ Results from multinomial logistic regression are reported (adjusted for clustering by respondent) as there was evidence that odds were not proportional

^6^ Results from random-effects logistic regression are reported for analyses with binary outcomes

^7^ The “don’t know” option was not included in the random-effects logistic regression analysis for this variable.

### Associations of casual interaction and semi-ownership human-cat relationships with interactions and caretaking behaviours displayed towards study cats

All interactions and caretaking behaviours were significantly more likely to be displayed towards cats in semi-ownership relationships compared to cats in casual interaction relationships (p<0.01) (Table 3).

**Table 3:** Distributions of study cats in semi-ownership and casual interaction human-cat relationships that had received various interactions and caretaking behaviours and associations between these and the human-cat relationship of the study cat.

Dependent variable and categories	Casual interaction cats n (% of cats)	Semi-owned cats	Odds ratio/relative risk ratio^1^	95% Confidence interval	P value^2^
		n (% of cats)			
**Held/stroked/cuddled cat (n = 446)** ^**3**^			21.1	12.1 to 36.6	**0.000**
Never or occasionally	319 (92) ^4^	35 (36) ^4^			
Sometimes	22 (6)	27 (28)			
Daily	7 (2)	36 (37)			
**Spent time with cat (n = 445)** ^**3**^			20.0	11.7 to 34.2	**0.000**
Never	312 (90)	32 (33)			
Sometimes	25 (7)	23 (24)			
Daily	10 (3)	43 (44)			
**Played with cat (n = 447)** ^**5**^					**0.000**
Never	240 (69)	30 (31)	Base outcome		
Occasionally	81 (23)	14 (14)	1.4	0.7 to 2.7	0.35
Sometimes	23 (7)	26 (27)	9.0	4.6 to 17.8	0.000
Daily	5 (1)	28 (29)	44.8	16.8 to 124.8	0.000
**Cat was confined to the respondent’s property (n = 445)** ^**3**^			7.6	3.7 to 15.7	**0.000**
Never	334 (96)	76 (78)			
Occasionally	6 (2)	6 (6)			
Sometimes	3 (1)	6 (6)			
Daily	4 (1)	10 (10)			
**Organized holiday care for cat (n = 448)** ^**5**^					**0.000**
Never	321 (92)	41 (42)	Base outcome		
Sometimes	22 (6)	14 (14)	5.0	2.4 to 10.5	0.000
Always	7 (2)	43 (44)	48.1	20.3 to 113.9	0.000
**Provided a scratching post for cat (n = 446)** ^**5**^					**0.000**
Never	332 (95)	65 (66)	Base outcome		
Occasionally	8 (2)	4 (4)	2.6	0.8 to 8.7	0.14
Sometimes	4 (1)	4 (4)	5.1	1.2 to 21.0	0.02
Always	4 (1)	25 (26)	31.9	10.8 to 94.8	0.000
**Provided a litter tray for cat (n = 446)** ^**3**^			11.7	5.7 to 24.0	**0.000**
Never	336 (97)	69 (70)			
Occasionally	2 (1)	7 (7)			
Sometimes	2 (1)	1 (1)			
Always	8 (2)	21 (21)			
**Provided toys for cat (n = 446)** ^**3**^			11.2	6.3 to 20.0	**0.000**
Never	325 (93)	55 (56)			
Occasionally	12 (4)	16 (16)			
Sometimes	5 (1)	8 (8)			
Always	6 (2)	19 (19)			
**Had cat checked by a veterinarian (n = 447)** ^**3**^			8.8	5.1 to14.9	**0.000**
Never	318 (91)	52 (53)			
Occasionally	20 (6)	28 (29)			
Regularly	11 (3)	18 (18)			
**Had cat vaccinated (n = 446)** ^**3**^			9.8	5.4 to 18.0	**0.000**
Never	328 (94)	61 (62)			
Occasionally	8 (2)	13 (13)			
Regularly	12 (4)	24 (25)			
**Gave cat deworming medication (n = 445)** ^**3**^			12.2	7.1 to 21.1	**0.000**
Never	320 (92)	47 (48)			
Occasionally	13 (4)	23 (24)			
Regularly	14 (4)	28 (29)			
**Applied flea/tick control to cat (n = 446)** ^**3**^			11.1	6.5 to 18.8	**0.000**
Never	317 (91)	46 (47)			
Occasionally	17 (5)	25 (26)			
Regularly	14 (4)	27 (28)			
**Cat had been microchipped (n = 438)** ^**6**^			5.4	2.6 to 11.6	**0.000**
Yes	13 (4)	18 (19)			
No	295 (80)	75 (77)			
Don’t know^7^	33	4			
**A tag had been put on cat with the respondent’s contact details (external identification) (n = 438)** ^**6**^			6.0	1.9 to 18.9	**0.0019**
Yes	336 (99)	89 (92)			
No	5 (1)	8 (8)			
**Cat had been sterilized (n = 451)** ^**6**^			4.4	2.2 to 8.6	**0.000**
Yes	35 (34)	46 (71)			
No	63 (64)	19 (29)			
Don’t know^7^	225	33			
**Cat had had kittens (n = 448)** ^**6**^			3.7	1.8 to 7.3	**0.0005**
Yes	30 (10)	16 (30)			
No	260 (90)	38 (70)			
Don’t know^7^	60	44			

^1^ Odds ratio estimates are reported for ordered logistic regression and random-effects logistic regression; these estimate the odds of any particular interaction or caretaking behaviour outcome category for a cat with a semi-ownership human-cat relationship compared to those cats with a casual interaction human-cat relationship. Relative risk ratio (RRR) estimates are reported for multinomial logistic regression analyses; these estimate the probability of the specified interaction or caretaking behaviour outcome category rather than the base outcome for cats with a semi-ownership human-cat relationship compared to a casual interaction human-cat relationship

^2^ Bolded values are overall likelihood ratio test p-values; non-bolded values are Wald p-values for the specific level, relative to the reference category

^3^ Results from ordered logistic regression as >2 categories for outcome and there was no evidence that odds are not proportional.

^4^ Total numbers of respondents differ between variables as not all respondents answered each question and, within variables, percentages do not always sum to 100% due to rounding

^5^ Results from multinomial logistic regression are reported as there was evidence that odds were not proportional

^6^ Results from logistic regression are reported for analyses with binary outcomes

^7^ The “don’t know” option was not included in the logistic regression analysis for this variable.

## Discussion

We began by defining four cat human-cat relationship categories: casual interactions, semi-ownership, ownership of a passively acquired cat and ownership of an actively acquired cat. Method of acquisition of a cat (passive versus active) is a strong determinant of perception of cat ownership, with actively-acquired cats likely almost always perceived as owned [14]. Accordingly we were interested in assessing determinants of cat ownership perception by comparing respondents who perceived themselves as the owner of a passively-acquired cat and respondents who did not perceive themselves as owners of a cat that they regularly fed or otherwise cared for over a reasonably long period, whom we termed semi-owners. We also explored effects of perceived ownership by comparing interactions and caretaking behaviours between these two groups.

Perception of cat ownership was associated with interactions and caretaking behaviours displayed towards study cats. While semi-owners displayed the same types of interaction and caretaking behaviours as owners, they did so less commonly than owners. The direction of causality underlying these observed associations could not be determined in our study, as the cross-sectional design measured cat ownership perception, interactions and caretaking behaviours at only one point in time. While our statistical models were based on the simplifying assumption that cat ownership perception causes interactions and caretaking behaviours, these relationships are likely to be dynamic and interactive, with complex interactions and feedback mechanisms between perception of cat ownership, interactions, caretaking behaviours, and perceived cat friendliness and health. For example, a friendly cat may be more likely to interact with a person, who may then interact with the cat for longer, become more attached, and provide more care for the cat, which may ultimately result in the cat becoming friendlier (and healthier) [31]. Over time, the person is likely to develop more of a sense of ownership for the cat (as investment of time, money and care is recognized to result in a feeling of ownership [32]). These complexities could be explored using longitudinal studies, with data collected repeatedly over time from the same respondents.

Although perception of cat ownership was positively associated with the provision of all measured interactions and caretaking behaviours, some owners of passively-acquired cats did not display all interactions and caretaking behaviours while some semi-owners did, indicating that perception of ownership is not the only determinant of interactions and caretaking behaviours. The types of interactions and caretaking behaviours displayed towards cats considered unowned (i.e. semi-owned cats and cats with which the respondent only interacted casually) were similar to those displayed to owned passively-acquired cats, varying from stroking and feeding the cat to “responsible” caretaking behaviours, such as sterilization. This is consistent with previous reports [2,5,6,31,33]. The proportions of owners in our study who displayed “responsible” caretaking behaviours were similar or slightly higher than those reported in other studies [34,35,36], although it is difficult to make direct comparisons as the methodologies differed.

Determinants of perception of cat ownership identified in semi-owned and passively-acquired owned cats included association time, attachment score, perceived cat friendliness and health, and psychosocial factors relating to feeding stray cats. Many determinants of cat ownership perception related to feelings about stray cats and the acceptability of feeding a stray cat. People who thought that feeding a stray cat made them feel good, and that people important to them would approve of them feeding a stray cat were more likely to be semi-owners rather than owners of passively acquired cats; people who said they could not feed a stray because of their beliefs or that strays were a nuisance were more likely to be owners. This is consistent with the Theory of Planned Behaviour [15,16]: those who believe it is socially acceptable and altruistic to feed a stray cat may not feel the need to take ownership of the animal. Conversely, a person who does not think it is acceptable to feed a stray cat will more likely feel the necessity to take ownership or not become involved at all. If semi-owners are more susceptible to normative social pressure as indicated by our data, this represents a potential point of intervention: their caretaking behaviours could be influenced by appropriate social marketing messages that reinforce the importance of sterilizing unowned cats.

Many cat semi-owners in this study were also cat owners, a phenomenon which has been previously described [6,37]. Although people who feed unowned cats are reported to genuinely care for the cats [11,33], the factors that prevent them from taking ownership of the cats that they semi-own are poorly understood. Perceived cat friendliness and health were positively related to perceived ownership in our study, indicating that absence of these cat attributes may create a barrier to semi-owners assuming ownership of a specific cat. It has been suggested that a person taking ownership of an unowned cat is unlikely if the cat is not friendly [2,11]. There may also be other reasons why a cat owner cannot or will not take ownership for a specific semi-owned cat. For example, there may be local government limits on the number of cats a person can legally own, or the semi-owned cat may not be socially compatible with an existing pet. Although many cat semi-owners also own cats, many cat owners in this study had only casual interactions with an unowned cat and yet did not have a relationship with that cat that could be classified as semi-ownership. Our study suggests that human-cat relationships differ due to factors relating to the individual cat and circumstances involved, as well as differing underlying psychology, which means some cat owners who have contact with an unowned cat will become a semi-owner and some will have only casual interactions.

In our study population, the median age and employment status of our sample was similar to Australian census data [30], but education levels and indicators of socioeconomic status were higher than the national average, likely because the internet survey participants have access to, and used, a computer. Other similar work has also reported respondents with higher than average socioeconomic status and education levels [2,17,18,19,21]. The population also consisted of more women than men, which may derive from the greater concern for animal welfare reported for women compared to men [38] and is consistent with other research in the area of pet ownership and human animal-bond [2,17,18].

In our study, 10% of respondents were semi-owners, which is lower than previous reports of semi-ownership in Australia [2].This disparity is understandable in light of our study populations’ demographic—highly educated people who had access to, and used, a computer and social media and who consequently might be expected to be exposed to media reports about cat overpopulation issues—and our revised definition of semi-ownership, which would have excluded many of the cats included in other studies. Nevertheless, cat semi-ownership was still occurring in this population. Semi-owners who feed unowned cats frequently or always, or who are more aware of media reports about the unwanted cat problem are reportedly more likely to sterilize their cats [6,33]. Indeed, 47% of the semi-owned cats in our study were known to be sterilized, as opposed to 20% found in previous Australian studies [2]. Despite the relatively high proportion of sterilised semi-owned cats in this study, these cats were, nevertheless, more likely to have produced kittens than the owned cats, and owned cats were significantly more likely to have been sterilized than semi-owned cats. These findings attest to the veracity of previous claims that semi-owned cats are likely to be contributing considerably to the unwanted cat problem [1,2,12], and indicate that more widespread public awareness of the negative impacts associated with semi-ownership is warranted.

Our study has implications for the potential for delivery of such public awareness and educational messages through highly accessible semi-owners (such as those in our study) to less accessible semi-owners. The use of “gatekeepers” to provide access to groups of hard-to-reach or socially excluded people in research is well recognized [39]. Semi-owners may be difficult to access because they may be distrustful of authorities involved with unwanted cat management [31,40,41] or concerned about social exclusion and criticism since feeding unowned cats may attract disapproval and censure from others [10]. Accessible semi-owners who participate in social media platforms (such as the respondents in our study) could be used as “gatekeepers” to facilitate education and provision of services (particularly cat sterilization) to other semi-owners. These “gatekeeper” semi-owners could also act as ambassadors for promoting responsible semi-ownership.

Human psychology research has identified that cognitive dissonance is a powerful driver of human behaviour, and explains why people often proceed with certain actions despite the knowledge that those actions may have negative outcomes [42,43,44]. For example, when informed about the negative consequences of cat semi-ownership, some semi-owners will accept this and change their behaviour to avoid the cognitive dissonance that is common when performing a behaviour that they believe to be wrong; they might take ownership of the cat, they might surrender the cat to a shelter or they might just stop feeding the cat. Other semi-owners may justify the behaviour through changing the dissonant cognition; for example, they might convince themselves that the evidence of negative consequences from cat semi-ownership is inconclusive. Other semi-owners will attempt to justify the behaviour by the addition of new cognitions; for example, they might focus on their love of cats and their perceived altruism toward cats “in need” and convince themselves that their actions are benevolent [10,45]. These concepts demonstrate why some people continue semi-ownership behaviours regardless of education about the negative impacts of this behaviour.

If the problems associated with cat semi-ownership are to be mitigated, it would be prudent to consider alternative options to those currently recommended, which is surrendering unowned cats to a shelter or municipal pound [46,47]. An alternative approach is to accept that some semi-ownership will continue despite educational campaigns to the contrary and to encourage and facilitate sterilization of these cats. This approach would require the revision and clarification of current cat classification systems in some jurisdictions to allow cats that have a human caretaker (including semi-owned cats) to be sterilized and remain with their semi-owner, even if the semi-owner cannot or will not take full “ownership”. Currently, this is not legal in those jurisdictions where a semi-owned cat is classified as a “feral” cat. Cat classification systems vary between jurisdictions, for example, some Australian states have a classification system that could be interpreted as allowing an approach such as that described, but others do not [48,49]. A consistent classification system is needed to facilitate cat management. Distinguishing between cats that are directly or indirectly dependent on humans and those that are not dependent on humans (feral cats) has been proposed in New Zealand [50].

The findings demonstrating that perception of cat ownership is strongly associated with both association time and caretaking behaviours suggest that, if semi-owners continue to care for the cat, it is possible that some will, over time eventually take ownership of the cat. In the U.S. it has been reported that 68% of people who found cats and were unable to find the owner ended up keeping the cat and assuming ownership for it [51]. However, caution is needed when extrapolating such findings to other countries and cultures. Companion animal ownership may be perceived differently in different cultures, as the interpretation of “owner” and “ownership” can vary under the influence of different legal, educational, cultural and religious constructs [5,52].

The definition of the four cat human-cat relationship categories used in this study to analyse determinants of cat ownership perception were based on previous literature [2,5,14,46] but the extent to which these categories represent reality in this novel area of research is unknown. Further work would be needed to validate the approach and categories used in this study. Quite possibly cat ownership perception may be best described on a continuum rather than in discrete categories as were used for the purposes of data analysis in this study.

Our revised definition of semi-ownership to include an association time of at least 1 month and frequent feeding resulted in a distinct demarcation between cats with semi-ownership relationships and casual interaction relationships. All interactions and caretaking behaviours were significantly more likely to be displayed towards cats in semi-ownership relationships compared to casual interaction relationships. This revised definition of cat semi-ownership may be useful for shelters, welfare and government agencies wanting to differentiate these two types of relationships in order to inform cat management strategies.

## Conclusions

This study has identified specific determinants of perception of cat ownership: association time, attachment score, cat factors, and feelings about “stray” cats and whether it was acceptable to feed a “stray” cat. Although both owners and semi-owners displayed the same types of interactions and caretaking behaviours towards cats, owners were more likely to display all interactions and caretaking behaviours than semi-owners. In addition, semi-owned cats were more likely to have had kittens than owned cats and in this way contribute to unwanted cat numbers. Preventing semi-ownership behaviour entirely is difficult for a variety of psychosocial reasons, but may not be essential to achieve the goals of improving cat care/welfare and reducing the number of unwanted kittens born. Encouraging and facilitating sterilization of semi-owned cats whose semi-owner cannot or will not take ownership of the cat may be an alternative and effective way to address the issues caused by cat semi-ownership. Our findings can inform policies and strategies aimed at mitigating the contribution of semi-owners to the unwanted cat problem, by providing a mechanism to distinguish semi-ownership from casual cat interactions, strategies to access semi-owners, and educational approaches to modify semi-ownership behaviour.

## Supporting Information

S1 Table Questionnaire categories and data variable details. (DOCX)

S2 Table Distributions of study cats in semi-ownership and ownership human-cat relationships and potential determinants of ownership perception that were not significantly associated with the perception of ownership of the study cat. (DOCX)
