# Supplementary material for: Cat Ownership Perception and Caretaking Explored in an Internet Survey of People Associated with Cats
Source: PLoS One. 2015 Jul 28;10(7):e0133293. doi: 10.1371/journal.pone.0133293 (PMC4517794; doi:10.1371/journal.pone.0133293)
Supplement: S1 Table — (DOCX) [file pone.0133293.s001.docx]

**Supporting Information Table S1: Questionnaire categories and data variable details**

| **Categories** | **Variable details** |
| --- | --- |
| Respondent demographics | Gender, age, residential location, postcode, occupation status (for example employed full-time, unemployed etc.), income and education level. |
| Beliefs about cats, cat ownership and “stray” cats | The respondent’s level of agreement^1^ with the statements: “cats are independent”, “cats are peaceful”, “cats carry disease”, “cats are friendly”, “cats are dirty”, “cats are good company” “cats kill wildlife”, “cats are expensive pets”, “cats make good pets”, “stray cats take care of themselves”, “stray cats spread disease”, and “stray cats are a nuisance”. |
| Attitudes towards cats, cat ownership and “stray” cats | The respondent’s level of agreement^1^ with the statements: “I like cats”, “owning a cat makes you happy”, “feeding a stray cat is the right thing to do”, feeding stray cats stops them from killing wildlife”, feeding a stray cat makes me feel good”, “I feel sorry for stray cats” and “stray cats are a problem”. |
| Social norms relating to cat ownership and “stray” cats | The respondent’s level of agreement^1^ with the statements: “People who are important to me would approve of me owning a cat” and “People who are important to me would approve of me feeding a stray cat”. |
| Perceived behavioural control relating to cat ownership and “stray” cats | The respondent’s level of agreement^1^ with the statements: “my feelings towards cats make me want to have a cat”, “I could have a cat in my accommodation”, “it would be difficult for me to have a cat with my other pets”, “my financial situation would make it difficult for me to have a cat”, “I could not have a cat because it would kill the local wildlife”, “I could not feed a stray cat because of my beliefs”, and “financially I could afford to feed a stray cat”. |
| Cat ownership | Did the respondent own a cat? |
|  | Did the respondent plan to get their cat? |
| Stray cat interactions | In the past 5 years had the respondent interacted with a stray cat(s)? |
| Cat factors | Cat sex, association time with the cat, cat’s health, behaviour and friendliness. |
| Attachment to the study cat | The adopter’s self-rated attachment to the cat (measured on a Likert scale of 1-5 where 1= not at all attached and 5 = very attached). |
| Interactions with the study cat | The frequency of the respondent’s interactions with the cat (holding/stroking/ cuddling the cat, spending time with the cat and playing with the cat)^2^ |
| Caretaking behaviours towards the study cat | The frequency of the respondent confining the cat to their property^2^ |
|  | The frequency of the respondent organising holiday care for the cat^3^ |
|  | The frequency of the respondent providing food and water, a scratching post, a litter tray and toys for the cat^4^ |
|  | The frequency that the respondent had had the cat checked by a veterinarian, vaccinated, gives the cat de-worming medication and applies flea/tick control to the cat^5^ |
|  | Had the cat been mircohipped?^6^ |
|  | Had the respondent put a tag on the cat with their contact details (external identification)? ^6^ |
|  | Had the cat been sterilised? ^6^ |
|  | Had the cat had kittens? ^6^ |

^1^ Responses to these questions were quantified using 5-point Likert scales measuring agreement with the statements, from strongly disagree to strongly agree with the middle category as “neither agree nor disagree”.

^2^ Response categories were never, occasionally, sometimes, and daily.

^3^ Response categories were never, sometimes, always.

^4^ Response categories were never, occasionally, sometimes, always.

^5^ Response categories were never, occasionally, regularly.

^6^ Response categories were yes or no.
