# Supplementary material for: Cat Ownership Perception and Caretaking Explored in an Internet Survey of People Associated with Cats
Source: PLoS One. 2015 Jul 28;10(7):e0133293. doi: 10.1371/journal.pone.0133293 (PMC4517794; doi:10.1371/journal.pone.0133293)
Supplement: S2 Table — (DOCX) [file pone.0133293.s002.docx]

**Supporting Information Table S2: Distributions of study cats in semi-ownership and ownership human-cat relationships and potential determinants of ownership perception that were not significantly associated with the perception of ownership of the study cat^1^**

| **Exposure Variable** | **Semi-owned cats**  **n (% of cats)** | **Owned passively-acquired cats**  **n (% of kittens)** | **Odds ratio^2^** | **95% Confidence interval** | **P value^3^** |
| --- | --- | --- | --- | --- | --- |
| **Respondent gender (n=342)** | | | | | **0.58** |
| Male | 8 (8) | 35 (14) | Reference category | | |
| Female | 90 (92) | 209 (86) | 0.7 | 0.3 to 1.7 | 0.46 |
| **Respondent age (n=340)** | | | | | **0.55** |
| 18-25 | 8 (8) | 28 (12) | Reference category | | |
| 26-35 | 28 (29) | 69 (29) | 0.5 | 0.2 to 1.4 | 0.22 |
| 36-45 | 28 (29) | 52 (22) | 0.3 | 0.1 to 1.0 | 0.04 |
| 46-55 | 19 (19) | 54 (22) | 0.5 | 0.2 to 1.3 | 0.14 |
| 56-65 | 13 (13) | 12 (14) | 0.5 | 0.1 to 1.4 | 0.17 |
| ≥66 | 2 (2) | 6 (3) | 0.2 | 0.0 to 1.7 | 0.15 |
| **Respondent residential location (n=341)** | | | | | **0.48** |
| Suburban | 47 (48) | 126 (52) | Reference category | | |
| Urban | 28 (29) | 48 (20) | 0.6 | 0.3 to 1.1 | 0.11 |
| Regional city | 6 (6) | 16 (7) | 0.9 | 0.3 to 2.8 | 0.89 |
| Country town | 6 (6) | 19 (8) | 1.2 | 0.4 to 3.4 | 0.80 |
| Rural | 6 (6) | 11 (5) | 0.5 | 0.2 to 1.7 | 0.29 |
| Semi-rural | 5 (5) | 23 (10) | 2.0 | 0.6 to 6.3 | 0.24 |
| **Index of relative socioeconomic advantage disadvantage decile (n=328)** | | | | | **0.88** |
| 1-2 | 9 (10) | 16 (7) | Reference category | | |
| 3-4 | 10 (11) | 30 (13) | 1.0 | 0.6 to 6.9 | 0.26 |
| 5-6 | 22 (23) | 46 (20) | 1.2 | 0.4 to 3.8 | 0.68 |
| 7-8 | 23 (24) | 60 (26) | 1.5 | 0.5 to 4.4 | 0.45 |
| 9-10 | 31 (33) | 81 (35) | 1.5 | 0.5 to 4n3 | 0.44 |
| **Respondent occupation status (n=342)** | | | | | **0.57** |
| Employed full time | 44 (45) | 119 (49) | Reference category | | |
| Employed part time | 9 (9) | 37 (15) | 1.5 | 6.3 to 3.7 | 0.36 |
| Casual worker | 5 (5) | 16 (6) | 0.7 | 0.2 to 2.1 | 0.47 |
| Homemaker | 7 (7) | 12 (5) | 0.6 | 0.2 to 1.6 | 0.28 |
| Student | 17 (17) | 21 (9) | 0.6 | 0.3 to 1.4 | 0.24 |
| Retired | 1 (1) | 11 (5) | 2.9 | 0.3 to 27.7 | 0.35 |
| Self-employed | 10 (10) | 16 (7) | 0.5 | 0.2 to 1.3 | 0.13 |
| Unemployed | 3 (3) | 6 (3) | 1.1 | 0.2 to 5.5 | 0.90 |
| Other | 2 (2) | 7 (32) | 1.4 | 0.2 to 9.4 | 0.71 |
| **Respondent income (n=338)** | | | | | **0.94** |
| Below average | 18 (19) | 44 (18) | Reference category | | |
| Average | 60 (62) | 139 (58) | 0.9 | 0.4 to 1.8 | 0.73 |
| Above average | 19 (20) | 58 (24) | 0.9 | 0.4 to 2.1 | 0.79 |
| **Respondent education level (n=338)** | | | | | **0.79** |
| Primary school or no formal schooling | 0 | 0 |  | | |
| Secondary school | 17 (17) | 40 (17) | Reference category | | |
| University undergraduate degree | 40 (41) | 76 (32) | 0.8 | 0.3 to 1.7 | 0.52 |
| University postgraduate degree | 25 (26) | 67 (28) | 1.0 | 0.4 to 2.2 | 0.94 |
| Technical or trades college | 11 (11) | 43 (18) | 1.5 | 0.6 to 3.9 | 0.45 |
| Other | 5 (5) | 14 (6) | 1.1 | 0.3 to 4.1 | 0.90 |
| **Beliefs about cats, cat ownership and “stray” cats** | | | | | |
| *Agreement with the statements:* | | | | | |
| *“Cats are independent” (n=342)* | | | | | **0.93** |
| Strongly disagree | 1 (1) | 2 (1) | Reference category | | |
| Somewhat disagree | 9 (9) | 26 (11) | 1.2 | 0.1 to 17.2 | 0.88 |
| Neither agree nor disagree | 8 (8) | 26 (11) | 1.5 | 0.1 to 21.1 | 0.77 |
| Somewhat agree | 45 (46) | 125 (51) | 1.4 | 0.1 to 17.1 | 0.81 |
| Strongly agree | 35 (36) | 65 (27) | 1.0 | 0.1 to 13.1 | 0.98 |
| *“Cats are peaceful” (n=341)* | | | | | **0.41** |
| Strongly disagree | 1 (1) | 1 (1) | Reference category | | |
| Somewhat disagree | 3 (3) | 17 (7) | 9.1 | 0.4 to 235.9 | 0.18 |
| Neither agree nor disagree | 23 (24) | 56 (23) | 2.9 | 0.1 to 60.5 | 0.49 |
| Somewhat agree | 47 (48) | 134 (55) | 3.6 | 0.2 to 72.2 | 0.41 |
| Strongly agree | 24 (25) | 35 (14) | 2.2 | 0.1 to 45.4 | 0.62 |
| *“Cats carry disease” (n=342)* | | | | | **0.93** |
| Strongly disagree | 29 (30) | 72 (30) | Reference category | | |
| Somewhat disagree | 30 (31) | 87 (36) | 1.0 | 0.5 to 1.9 | 0.95 |
| Neither agree nor disagree | 22 (23) | 48 (20) | 0.7 | 0.3 to 1.5 | 0.39 |
| Somewhat agree | 15 (15) | 34 (14) | 1.1 | 0.5 to 2.4 | 0.90 |
| Strongly agree | 2 (2) | 3 (1) | 0.9 | 0.1 to 6.8 | 0.93 |
| *“Cats are friendly” (n=342)* | | | | | **0.10** |
| Strongly or somewhat disagree | 1 (1) | 5 (2) | Reference category | | |
| Neither agree nor disagree | 9 (9) | 53 (22) | 1.1 | 0.1 to 13.3 | 0.96 |
| Somewhat agree | 63 (64) | 148 (61) | 0.4 | 0.0 to 4.7 | 0.48 |
| Strongly agree | 25 (26) | 38 (16) | 0.3 | 0.0 to 3.6 | 0.34 |
| *“Cats are dirty” (n=342)* | | | | | **0.79** |
| Strongly disagree | 58 (59) | 145 (59) | Reference category | | |
| Somewhat disagree | 28 (29) | 70 (29) | 1.0 | 0.6 to 1.8 | 0.98 |
| Neither agree nor disagree | 9 (9) | 21 (9) | 1.2 | 0.5 to 3.0 | 0.74 |
| Somewhat agree | 2 (2) | 3 (1) | 0.3 | 0.0 to 2.1 | 0.22 |
| Strongly agree | 1 (1) | 5 (2) | 2.7 | 0.3 to 28.6 | 0.42 |
| *“Cats are good company” (n=342)* | | | | | **0.25** |
| Strongly or somewhat disagree | 1 (1) | 1 (0) | Reference category | | |
| Neither agree nor disagree | 1 (1) | 10 94) | 18.8 | 0.5 to 773.6 | 0.12 |
| Somewhat agree | 27 (28) | 84 (34) | 2.1 | 0.2 to 83.8 | 0.36 |
| Strongly agree | 69 (70) | 49 (61) | 2.7 | 0.1 to 55.1 | 0.51 |
| *“Cats make good pets” (n=342)* | | | | | **0.79** |
| Strongly or somewhat disagree | 2 (2) | 4 (2) | Reference category | | |
| Neither agree nor disagree | 1 (1) | 3 (1) | 1.1 | 0.1 to 27.6 | 0.95 |
| Somewhat agree | 27 (28) | 91 (37) | 0.9 | 0.1 to 6.1 | 0.87 |
| Strongly agree | 68 (69) | 146 (60) | 0.6 | 0.1 to 4.3 | 0.63 |
| *“Stray cats take care of themselves” (n=342)* | | | | | **0.53** |
| Strongly disagree | 18 (18) | 31 (13) | Reference category | | |
| Somewhat disagree | 41 (42) | 78 (32) | 1/1 | 0.5 to 2.3 | 0.85 |
| Neither agree nor disagree | 20 (20) | 67 (28) | 1.6 | 0.7 to 3.7 | 0.28 |
| Somewhat agree | 16 (16) | 57 (23) | 1.9 | 0.8 to 4.5 | 0.17 |
| Strongly agree | 3 (3) | 11 (5) | 2.6 | 0.6 to 12.1 | 0.21 |
| *“Stray cats spread disease” (n=342)* | | | | | **0.32** |
| Strongly disagree | 4 (4) | 6 (3) | Reference category | | |
| Somewhat disagree | 25 (26) | 52 (21) | 0.8 | 0.2 to 4.1 | 0.83 |
| Neither agree nor disagree | 35 (36) | 74 (30) | 0.9 | 0.2 to 4.2 | 0.90 |
| Somewhat agree | 31 (32) | 97 (30) | 1.7 | 0.4 to 7.8 | 0.51 |
| Strongly agree | 3 93) | 15 (6) | 2.6 | 0.4 to 19/1 | 0.34 |
| **Attitudes towards cats, cat ownership and “stray” cats** | | | | | |
| *Agreement with the statements:* | | | | | |
| *“I like cats” (n=342)* | | | | | **0.67** |
| Did not agree^4^ | 2 (2) | 9 (4) | Reference category | | |
| Somewhat agree | 16 (16) | 49 (20) | 0.6 | 0.1 to 3.4 | 0.55 |
| Strongly agree | 80 (82) | 186 (76) | 0.5 | 0.1 to 2.5 | 0.37 |
| *“Owning a cat makes you happy” (n=342)* | | | | | **0.56** |
| Strongly or somewhat disagree | 1 (1) | 3 (1) | Reference category | | |
| Neither agree nor disagree | 4 (4) | 18 (7) | 2.8 | 0.2 to 40.0 | 0.46 |
| Somewhat agree | 22 (23) | 78 (32) | 1.7 | 0.2 to 19.7 | 0.67 |
| Strongly agree | 71 (73) | 145 (59) | 1.2 | 0.1 to 13.6 | 0.87 |
| *“I feel sorry for stray cats” (n=342)* | | | | | **0.06** |
| Strongly or somewhat disagree | 1 (1) | 16 (7) | Reference category | | |
| Neither agree nor disagree | 8 (8) | 22 (9) | 0.1 | 0.0 to 1.0 | 0.05 |
| Somewhat agree | 40 (41) | 115 (47) | 0.1 | 0.0 to 0.9 | 0.04 |
| Strongly agree | 49 (5) | 91 (37) | 0.1 | 0.0 to 0.6 | 0.02 |
| *“Stray cats are a problem” (n=342)* | | | | | **0.52** |
| Strongly disagree | 4 (4) | 9 (4) | Reference category | | |
| Somewhat disagree | 7 (7) | 12 (5) | 0.7 | 0.1 to 4.0 | 0.72 |
| Neither agree nor disagree | 20 (20) | 43 (18) | 0.8 | 0.2 to 3.2 | 0.70 |
| Somewhat agree | 49 (50) | 110 (45) | 1.0 | 0.2 to3.7 | 0.94 |
| Strongly agree | 18 (18) | 70 (29) | 1.7 | 0.4 to 6.9 | 0.49 |
| **Social norms relating to cat ownership and “stray” cats** | | | | | |
| *Agreement with the statements:* | | | | | |
| *“People who are important to me would approve of me owning a cat” (n=342)* | | | | | **0.47** |
| Strongly disagree | 1 91) | 4 (2) | Reference category | | |
| Somewhat disagree | 2 (2) | 6 (3) | 0.4 | 0.0 to 7.5 | 0.52 |
| Neither agree nor disagree | 4 (4) | 25 (10) | 1.3 | 0.1 to 20.1 | 0.84 |
| Somewhat agree | 31 (32) | 92 (38) | 0.6 | 0.1 to 7.9 | 0.72 |
| Strongly agree | 60 (61) | 117 (48) | 0.4 | 0.0 to 5.4 | 0.52 |
| **Perceived behavioral control relating to cat ownership and “stray” cats** | | | | | |
| *Agreement with the statements:* | | | | | |
| *“My feelings towards cats make me want to have a cat” (n=342)* | | | | | **0.17** |
| Strongly disagree | 1 (1) | 5 (2) | Reference category | | |
| Somewhat disagree | 3 (3) | 17 (7) | 0.7 | 0.1 to 10.1 | 0.78 |
| Neither agree nor disagree | 7 (7) | 32 (13) | 1.0 | 0.1 to 12.3 | 0.99 |
| Somewhat agree | 26 (27) | 72 (30) | 0.4 | 0.0 to 4.4 | 0.45 |
| Strongly agree | 61 (62) | 118 (48) | 0.3 | 0.0 to 3.4 | 0.31 |
| *“I could have a cat in my accommodation” (n=342)* | | | | | **0.08** |
| Strongly disagree | 6 (6) | 3 (1) | Reference category | | |
| Somewhat disagree | 5 (5) | 5 (2) | 3.1 | 0.4 to 22.8 | 0.27 |
| Neither agree nor disagree | 2 (2) | 3 (1) | 1.6 | 0.2 to 17.4 | 0.70 |
| Somewhat agree | 23 (24) | 90 (37) | 8.5 | 1.8 to 40.7 | 0.01 |
| Strongly agree | 62 (63) | 143 (59) | 5.0 | 1.1 to 22.8 | 0.04 |
| *“I could not have a cat because it would kill the local wildlife” (n=342)* | | | | | **0.08** |
| Strongly disagree | 53 (54) | 88 (36) | Reference category | | |
| Somewhat disagree | 33 (34) | 117 (48) | 2.2 | 1.3 to 4.0 | 0.01 |
| Neither agree nor disagree | 10 (10) | 30 (12) | 2.0 | 0.8 to 4.8 | 0.12 |
| Somewhat or strongly agree | 2 (2) | 9 (4) | 2.9 | 0.5 to 15.5 | 0.22 |
| *“It would be difficult for me to have a cat with my other pets” (n=342)* | | | | | **0.38** |
| Strongly disagree | 41 (42) | 85 (35) | Reference category | | |
| Somewhat disagree | 24 (25) | 97 (40) | 2.1 | 1.1 to 3.9 | 0.03 |
| Neither agree nor disagree | 22 (23) | 42 (17) | 1.3 | 0.6 to 2.6 | 0.53 |
| Somewhat agree | 10 (10) | 17 (7) | 0.8 | 0.3 to 2.2 | 0.69 |
| Strongly agree | 1 (1) | 3 (1) | 1.2 | 0.1 to 13.1 | 0.88 |
| *“My financial situation would make it difficult for me to have a cat” (n=342)* | | | | | **0.43** |
| Strongly disagree | 43 (44) | 88 (36) | Reference category | | |
| Somewhat disagree | 31 (32) | 113 (46) | 1.7 | 0.9 to 3.0 | 0.10 |
| Neither agree nor disagree | 13 (13) | 29 (12) | 1.3 | 0.5 to 2.9 | 0.60 |
| Somewhat agree | 9 (9) | 10 (4) | 0.7 | 0.2 to 2.1 | 0.50 |
| Strongly agree | 2 (2) | 4 (2) | 0.5 | 0.1 to 3.0 | 0.43 |
| **Cat demographics** | | | | |  |
| *Cat sex (n=327)* | | | | | **0.79** |
| Male | 43 (52) | 116 (48) | Reference category | | |
| Female | 40 (48) | 128 (53) | 1.1 | 0.6 to 1.9 | 0.71 |
| *Frequency of bad or annoying behaviour from the cat (n=342)* | | | | | **0.38** |
| Never | 43 (44) | 87 (36) | Reference category | | |
| Occasionally | 4 (4) | 25 (10) | 3.0 | 0.9 to 10.0 | 0.07 |
| Frequently | 50 (51) | 131 (54) | 1.1 | 0.6 to 1.9 | 0.74 |
| Always | 1 (1) | 1 (0) | 0.3 | 0.0 to 6.0 | 0.44 |

^1^ Variables that had an overall p-value of >0.05 in the initial screening are reported in this table. These results are those from the initial screening analyses.

^2^ Odds ratio estimates were adjusted for association time. Odds ratios refer to the odds of a cat having an ownership human-cat relationship compared to a semi-ownership human-cat relationship.

^3^ Bold values are overall likelihood ratio test p-values for variable; non-bolded values are Wald p-values for the specific category, relative to the reference category. All p-values have been adjusted using the Benjamini-Hochberg step-up FDR method.
